# Role of Influenza A virus protein NS1 in regulating host nuclear body ND10 complex formation and its involvement in establishment of viral pathogenesis

**DOI:** 10.1371/journal.pone.0295522

**Published:** 2024-01-02

**Authors:** Ujjal Das, Mamta Chawla-Sarkar, Swati Roy Gangopadhyay, Sanjit Dey, Rakhi Dey Sharma

**Affiliations:** 1 Barrackpore Rastraguru Surendranath College, Barrackpore, India; 2 Endocrine Research Facilities, Department of Animal Science, Rutgers University, New Brunswick, New Jersey, United States of America; 3 Division of Virology, National Institute of Cholera and Enteric Diseases, Beliaghata, Kolkata, India; 4 Department of Physiology, University of Calcutta, Kolkata, India; 5 Natural Science Research Centre of Belda College under Vidyasagar University and Department of Physiology, Belda College, Belda, Paschim Medinipur, India; University of South Florida, UNITED STATES

## Abstract

Influenza viral infection is a seasonal infection which causes widespread acute respiratory issues among humans globally. This virus changes its surface receptor composition to escape the recognition process by the host’s immune cells. Therefore, the present study focussed to identify some other important viral proteins which have a significant role in establishment of infection and having apparent conserved structural composition. This could facilitate the permanent vaccine development process or help in designing a drug against IAV (influenza A virus) infection which will eliminate the seasonal flu shot vaccination process. The NS1 (Non-structural protein 1) protein of IAV maintains a conserved structural motif. Earlier studies have shown its significant role in infection establishment. However, the mechanism by which viruses escape the host’s ND10 antiviral action remains elusive. The present study clearly showed that IAV infection and NS1 transfection in A549 cells degraded the main component of the ND10 anti-viral complex, PML and therefore, inhibited the formation of Daxx-sp100-p53-PML complex (ND10) at the mid phase of infection/transfection. PML degradation activated the stress axis which increased cellular ROS (reactive oxygen species) levels as well as mitochondrial dysfunction. Additionally, IAV/NS1 increased cellular stress and p53 accumulation at the late phase of infection. These collectively activated apoptotic pathway in the host cells. Along with the inactivation of several interferon proteins, IAV was found to decrease p-IKKε. A549 cells transfected with pcDNA3.1-NS1 showed a similar effect in the interferon axis and IKKε. Moreover, NS1 induced the disintegration of the host’s ND10 complex through the changes in the SUMOylation pattern of the PML nuclear body. These findings suggest the possible mechanism of how NS1 helps IAV to establish infection in the host cells. However, it demands further detailed study before targeting NS1 to develop permanent vaccines or novel drugs against IAV in future.

## Introduction

Mammals, including humans, have a well-developed immune system to combat any viral invasion. However, the seasonal spreading of influenza virus infection is still a major threats to its wide range of hosts. Seasonal influenza causes 16.7 deaths per 100,000 persons in the United States and approximately 500,000 deaths worldwide [1, 2]. In addition, Influenza A viruses have caused pandemics in the last century [3, 4]. The emerging evidence suggests that a seasonal flu is usually caused by the strain which has distinct antigenicity due to the accumulated mutations and is not recognized by antibodies elicited during past infection [5, 6]. In addition, viruses including IAVs evolve to exploit the host cellular apparatus that help them to complete their replication process and cope with the various antiviral defence systems of the hosts’ cells as well. Along with many other host proteins, a sub-nuclear structure known as Nuclear Domain -10 (ND-10) and its components have also been known to play role in antiviral defence in Influenza A virus infection [7]. ND10 has been identified as a frequent target site for a variety of different viruses during infection. These are dynamic cellular structures consisting of many transiently and permanently localized proteins [8]. Most of the studies have focused on nuclear-replicating DNA viruses. However, there are limited reports showing the replication of RNA viruses influenced by this nuclear substructure. The major components of ND10 or PML-NBs are PML (promyelocytic leukemia protein), hDaxx, Sp100 (speckled protein of 100 kDa), SUMO-1 (small ubiquitin-related modifier 1), and the Bloom syndrome helicase, BLM [8].

Recent studies have shown 18 different subtype of HA (hemagglutinin) and 11 subtypes of NA (neuraminidase) in IAV. IAV synthesizes various non-structural proteins (NSPs), such as NS1 (Non-Structural protein 1), which are only expressed in the infected host cells but are not packed in viral particle. The other NSPs are PA-X, PA-N155, PA-N182, M42, PB1 frame 2, PB1-N40, and NS3. NSPs have crucial roles in virulence, pathogenicity, and host defense suppression [9]. NS1 is a highly conserved, multifunctional protein of IAV known to affect the ND10 components formation in the nucleus [8].

Detailed study of this protein showed four different structural regions: two globular domains, a linker region, and a C-terminal disordered tail. In addition to two nuclear localization signals (NLSs) and one nuclear export signal (NES) have also been identified in NS1 [10]. NS1 has been found to be located inside the nucleus mainly and is transported to the cytoplasm at the later stage of infection [11]. It is also known to be involved in regulation of splicing, export and translation of viral mRNA which antagonizes host defence mechanisms [12]. The NS1 protein functions through the interaction with multiple hosts and viral cellular proteins [13, 14]. A variety of host proteins have been identified which interact with NS1 and NS2 [15, 16]. NS1, NS2 and M1 viral protein can interact with host’s ND10 complex [17]. Sato et al. (2003) showed the colocalization of M1 protein with PML-NB of ND 10 complex. They also observed the colocalization of NS1 and NS2 with M1. All these findings suggest the possible interaction of both the NS1 and NS2 with the host’s ND10 to escape the antiviral responses in host cells [18].

PML-NBs of ND10 complex are involved in the innate immune response of cells against viral invasion which is mediated by Interferon (IFN). We have explored different pathways to study the involvement of viral protein NS1 in escaping host’s antiviral mechanisms through the disruption of ND10 complex to establish viral pathogenesis. We ectopically expressed recombinant clone of NS1 in A549 cells to confirm the results obtained by the infection of A549 cells with IAV (PR8). We assessed the alteration in different components of ND10 in the presence of NS1 protein of Influenza A virus (PR8). Both immunoblot analysis and confocal microscopy confirmed the significant reduction in the formation of ND10 in the nucleus of both infected and transfected A549 cells. Our study also confirmed that 2/3 SUMOylation of PML might be one of the reasons for degradation of PML which subsequently led to decrease in functional ND10 complex formation in the infected and NS1 transfected A549 cells.

## Materials and methods

### Cell lines and virus propagation

Human pulmonary type II malignant epithelial cells (A549) and Madin-Darby Canine Kidney (MDCK) cells were purchased from the American Type Culture Collection (ATCC, Manassas, VA, USA). The cells were maintained according to the ATCC guidelines. A/Puerto Rico/8/34 (PR8; H1N1) viruses were propagated in MDCK cells in the presence of 1 μg/ml of l-1-tosylamide-2-phenylethyl chloromethyl ketone- (TPCK) treated trypsin (Sigma Aldrich, St. Louis, MO). A549 cells were cultured in Dulbecco’s Modified Eagle’s high glucose-containing medium (DMEM), and MDCK was maintained in Minimal Essential Medium. Both the media were supplemented with 10% foetal bovine serum (FBS) and 1% penicillin streptomycin solution. Cells were placed in an incubator with an atmosphere of 95% air and 5% CO_2_ in a 37 °C humidified incubator.

### Virus infection

Cells were washed with phosphate-buffered saline (PBS) and infected with Influenza A Virus H1N1 (A/Puerto Rico/8/34 i.e. PR8 virus) at the multiplicity of infection (MOI)) of 5 for 1 h at 37°C. After a 1 h adsorption, the inoculum was removed, and cells were incubated with DMEM containing 1% FBS (for A549 cells) or MEM containing 1% FBS (for MDCK) for the indicated time points [17].

### MTT assay

3-(4, 5-dimethylthiazolyl-2)-2, 5-diphenyltetrazolium bromide (MTT) assay was performed to assess viability. The assay was performed independently 3 times (technical replicate n = 3) and each time 3 replicates for one condition was taken (biological replicate n = 3). The cells (10^4^) were seeded in 96-well plate and incubated overnight for cell seeding. After infection, 10 μl of MTT (5 mg/ml) was added to each well at the indicated time points. Cells were incubated for 3 h at 37 °C in 5% CO_2_ pressure. The medium was discarded, and the purple formazan crystals were adequately dissolved with 100 μl of DMSO for 10 min on a rocker shaker. The absorbance was measured at 570 nm using an ELISA reader. The cell viability was calculated according to: OD sample/OD control × 100% [19].

### Nuclear morphology assay using DAPI staining

For the detection of changes in nuclear morphology after viral infection, the cells were stained with DAPI. After 24 h of infection, the cells were washed twice with phosphate buffered saline, fixed with 4% paraformaldehyde and permeabilized with 0.2% Triton X-100 and incubated with 1.0 μg/ml 4’,6-diamidino-2-phenylindole (DAPI). Fluorescent microscopy images were obtained using the EVOS fluorescence microscope (life technologies) [19].

### Acridine Orange/Ethidium Bromide (AO/EB) staining to detect apoptosis

Acridine Orange/Ethidium Bromide (AO/EB) staining was used to visualize the viability, alteration in membrane permeability and apoptotic body formation that are the characteristic of apoptosis. Cells were viewed and counted under a fluorescence microscope to quantify apoptosis. 100 μl of cell suspension (0.5 × 10^6^ to 2.0 × 10^6^ cells/ml) were incubated with 1 μl of AO/EB solution and mixed well just prior to microscopy and quantification. 10 μl of cell suspension was placed onto a microscopic slide covered with a glass coverslip. The cells were examined in EVOS fluorescence microscope using a fluorescein filter and a 60X objective [20].

### Measurement of intracellular ROS generation

The intracellular ROS was detected by flow cytometry using H_2_DCFDA. The A549 cells were harvested after 18 h of infection to measure intracellular ROS level. Cells were incubated with H_2_DCFDA (3 mM) for 10 min and the fluorescence was measured in FL-1 channel of BD FACS Calibur (Becton Dickinson, NJ, US) flow cytometer equipped with FlowJo 8.0 software (Version 8) [21].

### Measurement of externalization of phosphatidylserine (PS) in the outer leaflet of cell membrane

The flipping of PS in the outer leaflet of the cell membrane was detected by annexin V-APC and membrane damage was assessed by PI staining. After seeding of 1 × 10^6^ cells in culture plate, the infection was done as described previously. After 18 h of infection the cells were collected by trypsinization and incubated with annexin V-APC and PI staining solution for 15 min at room temperature. The fluorescence of 10,000 cells from different experimental groups were measured using a flow cytometer and data were analysed using FlowJo 8.0 software (Version 8) [19].

### Measurement of mitochondrial membrane potential (MMP)

JC-1 normally forms aggregates in mitochondria (590 nm emission; orange color) and comes out to cytosol upon alteration of ΔΨm (membrane potential). To identify the alteration in membrane potential, 1 × 10^6^ cells were seeded, and infection was given. After 18 h, the cells were trypsinized and incubated with JC-1 (2.5 μg/mL) for 20 min at room temperature. The fluorescence of 10^4^ cells was assessed using a flow cytometer at the emission of 525 and 590 nm. The data were analyzed using the FlowJo 8.0 software (Version 8) [19].

### Transient transfection

Lipofectamine-2000 was employed for the transient transfections of the recombinant pcDNA3.1 (expression vector) with or without NS1 insert in A549 cells. Approximately 1 ×10^6^ cells were transfected with 2 μg of H1N1/NS1 or control vector in a 6-well plate by Lipofectamine 2000 DNA Transfection Reagent following the protocol provided by the supplier. Cells were then collected 4, 16 and 48 h post-transfection by trypsinization, after washing with phosphate-buffered saline (PBS) [22].

### Immunoblot assay

For the analysis of protein expression, equal amounts of protein (50 μg) from different cell lysates were loaded in 10% sodium dodecyl sulfate-polyacrylamide gel electrophoresis (SDS-PAGE). The proteins were transferred to the activated PVDF membrane after electrophoresis. The membrane was blocked overnight at 4 °C with 5% bovine serum albumin (BSA) solution. Immunoblotting was done using monoclonal antibodies to Daxx, PML, NS1, p-IRF-3, p-STAT1, p-IKKε, p53 and sp100. β-actin and Histone 3 (H3) antibodies were used as loading control for cytosolic and nuclear extracts respectively. After incubation with the primary antibody for 3 h at room temperature, the secondary antibody tagged with horseradish peroxidase (HRP) was added and incubated for 2 h at room temperature. Protein bands were visualized using chemiluminescence kit. The relative protein levels were calculated by normalization to the amount of internal control proteins and were analyzed using GS-700 imaging densitometer and Molecular Analyst software (version 1.5, Bio-Rad Laboratories, Hercules, CA, USA) [23].

### Confocal microscopy

The cells were fixed with 4% paraformaldehyde at 4°C and then permeabilized with 0.2% Triton X-100, followed by blocking solution (2% BSA, and 0.1% Triton X-100 in PBS) addition for 1 h. The slides were incubated separately with PML, sp100, Daxx, p53, p-IRF-3, p-IKKε, p-STAT1, NS1, Sumo-1, Sumo-2/3 and isotype IgG primary antibody at a ratio of 1:250 each in the 1% BSA solution. After washing, incubation with Alexa488-labelled anti-mouse, and Alexa 488/647 labelled anti-rabbit antibodies at a ratio of 1:250 each in the blocking solution was performed. The slides were washed, covered with mounting solution, and visualized under a confocal microscope [24].

### Analysis of protein expression by flowcytometry

The cells were harvested at indicative time points after infection and fixed by 4% paraformaldehyde in PBS (pH 7.4). The cells were incubated with 0.1% Triton X-100 in PBS for 5 min for permeabilization. After washing twice in PBS containing 3% FBS, the permeabilized cells were incubated with primary antibodies (p-PI3K, p-Akt, p-Nrf2, and active caspase 3) for 2 h in ice followed by wash in PBS for two times. The cells were then incubated with either goat anti rabbit or rabbit anti mouse secondary antibody (depending upon the primary antibody) tagged with FITC fluorescence for 30 min in ice. The cells were washed with PBS for two times to remove the excess unbound secondary antibody. 10^4^ cells for each group were acquired and analyzed by BD FACS calibur equipped with FlowJo 8 (version 8). The expression of different proteins was checked for 3 independent times (n = 3) to calculate mean and SEM [19].

### Statistical analysis

Graph Pad Prism (version 6) software was used for statistical analysis of data. Data are presented as mean ± SEM. The significance between infection/transfection group and control was assessed using unpaired t-test/one-way ANOVA. Post-hock analysis for one-way ANOVA was done by the Newman-Keuls post hoc test. P <0.05 was considered significant. Additional details regarding quantitation and statistical analysis are provided in the figure legends. For cell culture studies where treatments were given, the experiments were repeated three times. The microscopy experiments were repeated five times. All the individual data points were present in S1 Table. The significance of differences was designated by ‘*’ between the control vs infection/transfection group.

## Results

### IAV infection reduced PML nuclear body structure in the host nucleus

**Fig 1:**
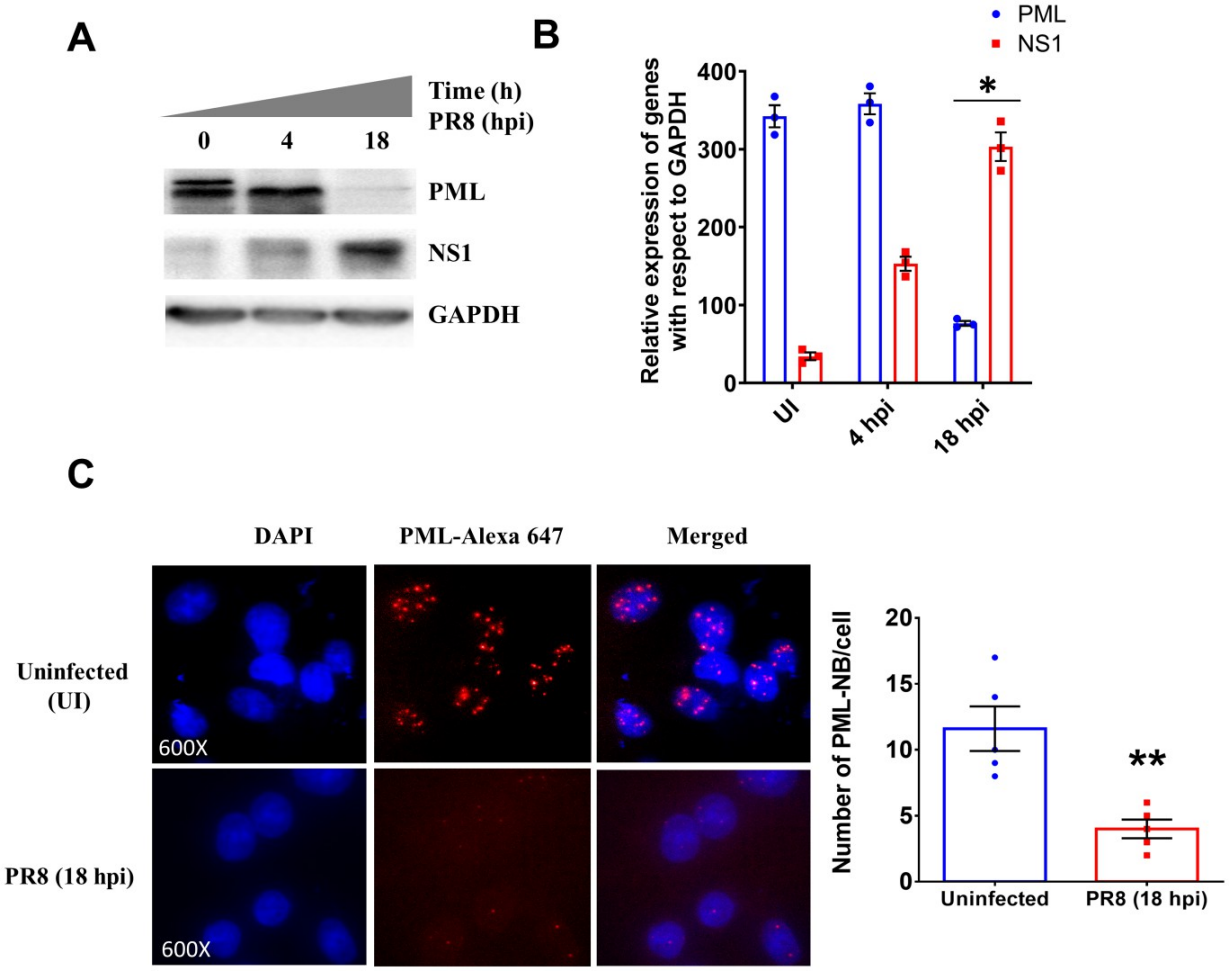
Degradation of PML nuclear body structure after IAV infection. **A.** The immunoblot images show the expression of different proteins. **B.** The densitometric analysis of immunoblot data. **C.** The confocal images show the degradation of the PML nuclear body after IAV infection. Nuclei were stained by DAPI and PML was stained with Alexa-647. **D.** The bar diagram shows the number of PML-NB/cell in uninfected and infected cells. The values represent the Mean±SEM of five microscopic fields of independent experiment. p < 0.05 was considered as significant. The values were represented as Mean±SEM [(n = 3 for immunoblot) and (n = 5 for microscopic data)]. The significance of differences was calculated between the ‘*’ control vs infection/transfection group.

Though several studies have been performed to find out the mechanism of how influenza virus established infection, there is no evidence showing how IAV infection modulates ND10 complex. As PML is one of the major components of the ND10 complex, we studied the effect of IAV infection on the PML nuclear body. IAV infected A549 cells showed minor change in expression of PML at the initial time point; however, it showed significant differences at late phase of infection (Fig 1A and 1B). The confocal microscopy data clearly indicated the degradation of the PML nuclear body in infected cells (Fig 1C and 1D). It is worthy to mention here that the expression of PML decreased gradually with the time while the virus infected cells showing rise in NS1 expression (Fig 1A and 1B).

### Effect of NS1 overexpression on PML, Daxx, p53, and sp100 of host ND10 component proteins

**Fig 2:**
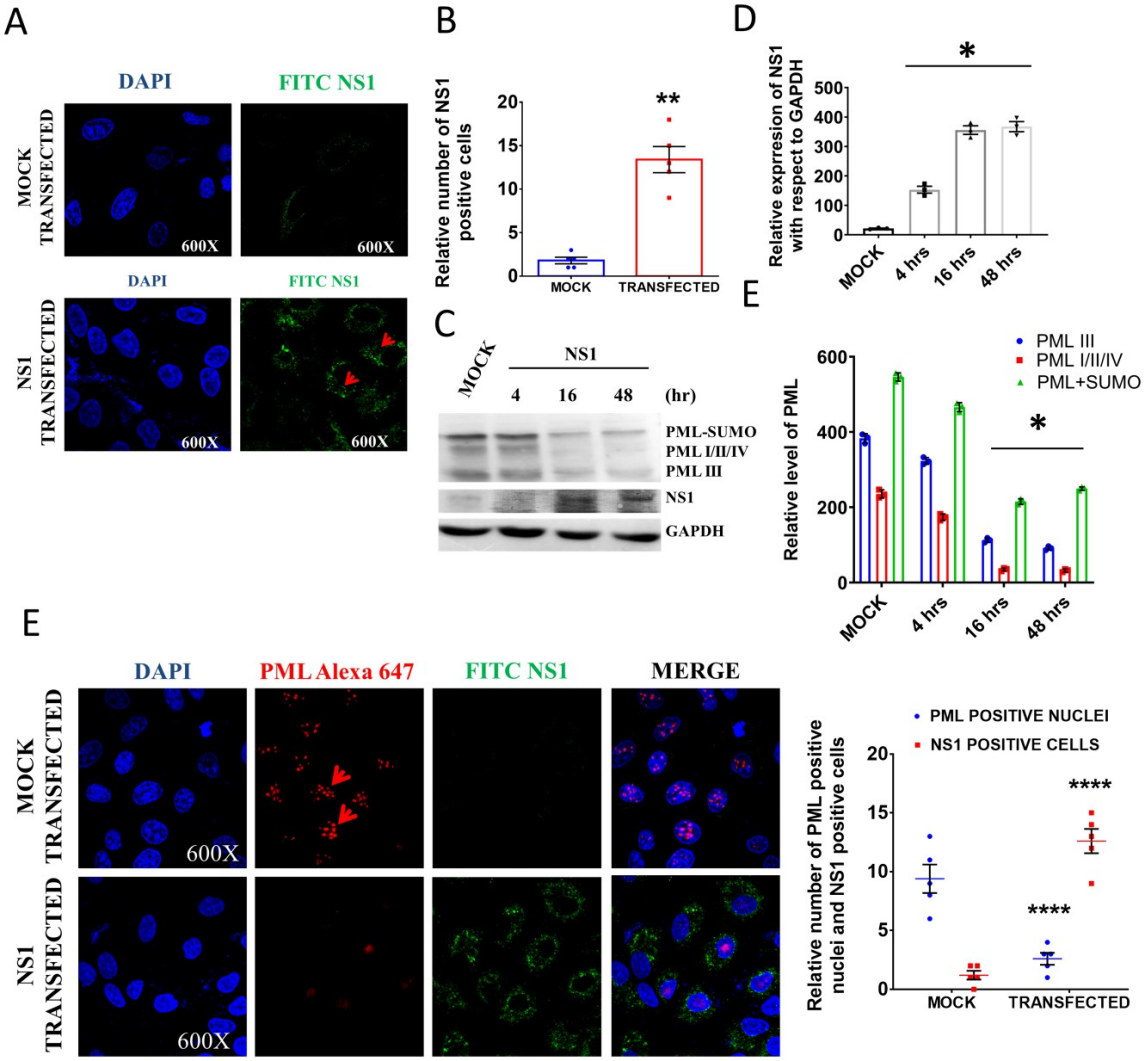
NS1 transfection degrades PML nuclear body structure. **A.** Expression of NS1 after transfection in A549 cells. DAPI was used to stain the nuclei and NS1 was stained by FITC tagged secondary antibody. **B.** Quantitative analysis of microscopic data represents the relative number of NS1 positive cells after transfection five microscopic fields of independent experiment **C.** Immunoblot images showed the PML expression at different time points after NS1 transfection. **D.** The densitometric analysis of immunoblot data. **E.** The fluorescence image showed the degradation of the PML nuclear body in NS1 transfected A549 cells. Nuclei were stained by DAPI and PML was stained by Alexa-647 (red) tagged secondary antibody. The red dots in the nuclei of mock transfected cells indicate the presence of the PML nuclear body. The bar diagram shows the number of PML-NB/cell in mock and NS1 transfected cells. The values represent the Mean±SEM [(n = 3 for immunoblot) and (n = 5 for microscopic data)]. p < 0.05 was considered as significant. The significance of differences was calculated between the ‘*’ control vs infection/transfection group.

As our results showed that IAV infection disrupted PML nuclear body when NS1 protein was expressed (Fig 1A and 1B), we cloned NS1 gene of IAV in pcDNA 3.1 expression vector and transfected into the A549 cells to ascertain the underlying mechanism. The transfection of NS1 in A549 cells was confirmed by confocal microscopy where the green fluorescence indicated the expression of NS1 in the transfected cells which was not observed in the mock transfected cells (Fig 2A and 2B). With the increase of NS1 expression after transfection, PML expression gradually decreased and after 48 h of transfection, it showed the maximum decrease as observed by the immunoblot data (Fig 2C and 2D). This data suggested the involvement of NS1 in downregulating the PML nuclear body. Furthermore, confocal microscopy images indicated the degradation of the PML nuclear body after NS1 transfection (Fig 2E) which corroborated with results obtained in the infection (18 hpi) model. As there is no significant change observed in NS1 expression after 16 and 48 h of transfection, we selected 16 h post transfection time point for the rest of the study.

**Fig 3:**
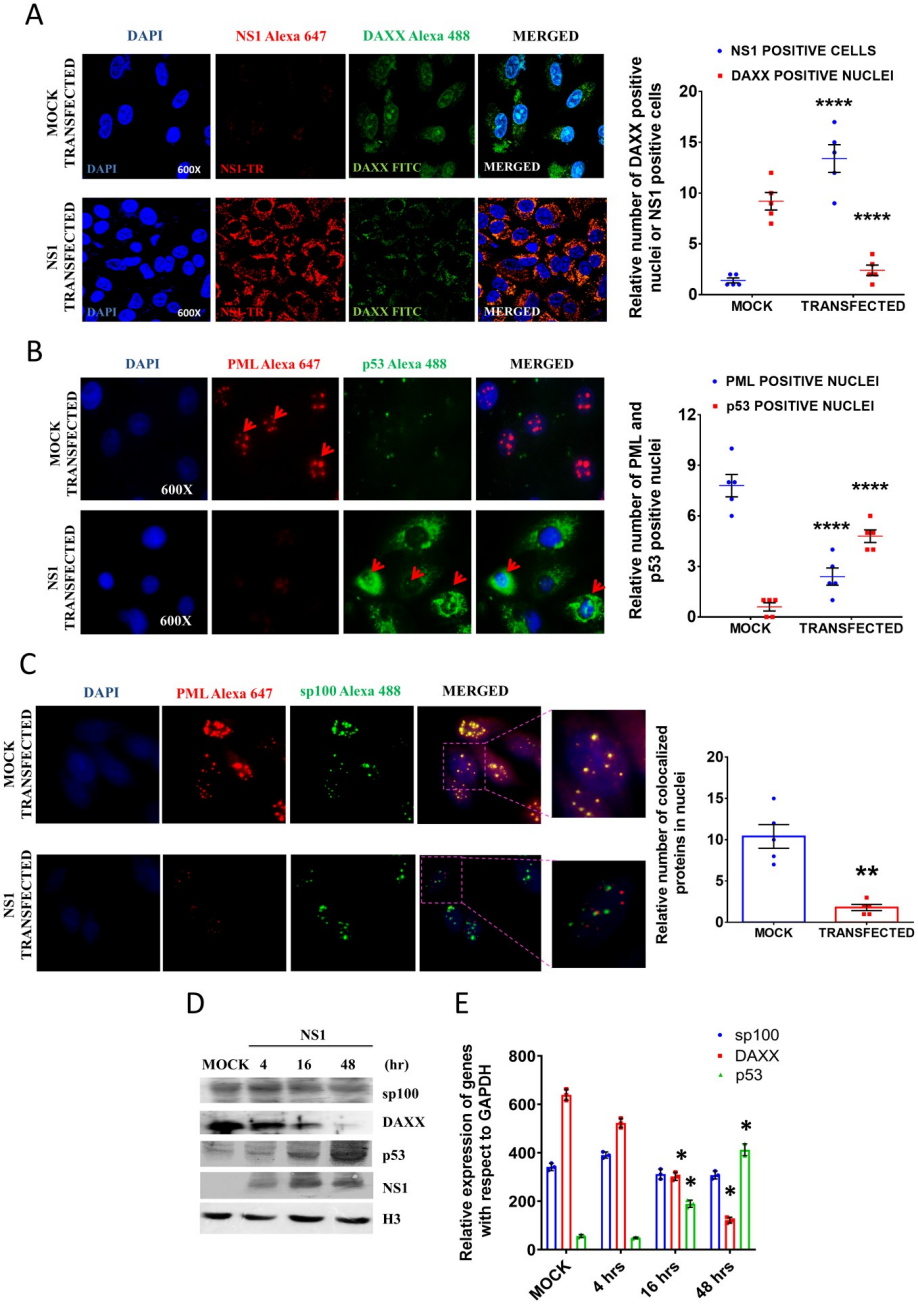
Effect of NS1 transfection on localization of Daxx, p53 and sp100. **A.** The confocal images show the subcellular localization of Daxx protein. The nuclei were stained by DAPI and Daxx was stained by Alexa-488 tagged secondary antibody and NS1 was stained by Alexa-647 tagged secondary antibody. The bar diagram represents the number of Daxx positive nuclei in mock and NS1 transfected cells. **B.** The localization of p53 protein in the PML nuclear body under mock and NS1 transfected conditions. P53 was stained by Alexa-488 tagged secondary antibody and PML was stained by Alexa 647 tagged secondary antibody. The red arrow indicated nuclear localization of p53 and PML. The bar diagram shows the number of PML-NB/cells and p53 positive nuclei in mock and NS1 transfected cells. **C.** The localization of sp100 protein in the PML nuclear body under mock and NS1 transfected conditions. Sp100 was stained by Alexa-488 tagged secondary antibody and PML was stained by Alexa 647 tagged secondary antibody. The yellow dots in the merged image indicate the co-localization of sp100 protein in the PML nuclear body. The bar diagram shows the number of PML-NB/cells and sp100 colocalized nuclei in mock and NS1 transfected cells. **D.** The immunoblot images and **E.** densitometric analysis showed time dependent expression of Daxx, p53 and sp100 proteins (nuclear fraction) in NS1 and mock transfected cells. p < 0.05 was considered as significant. The values were represented as Mean±SEM [(n = 3 for immunoblot) and (n = 5 for microscopic data)].

As ND10 contains sp100, Daxx and p53 along with PML, the expression and subcellular localization of these three other important components were checked (at the same time point) upon NS1 transfection. The subcellular localization of Daxx in presence or absence of NS1 was evaluated by the confocal microscopy (Fig 3A). After 16 h of NS1 transfection, most of the Daxx protein was found in cytosol which was otherwise mostly present within the nuclei of mock transfected cells. Therefore, in addition to the degradation of the PML nuclear body, NS1 inhibited nuclear translocation of Daxx in A549 cells (Fig 3A). The p53 protein plays a crucial role in the regulation of cell cycle progression and is a prime molecule for making the decision of programmed cell death. As a component of apoptotic pathway, cellular localization of p53 had been studied in mock and NS1 transfected A549 cells by confocal microscopy at later time points (after 48 h of transfection). In comparison to mock transfected cells, the level of p53 was upregulated in NS1 transfected cells (Fig 3B). However, p53 level was low/minimum after 4 h of transfection. Previous literature showed the accumulation of p53 after 24 h of IAV infection [25]. It has been reported that both the IAV infection and NS1 transfection inhibits ubiquitin ligase RNF43 and lead to the accumulation of p53 after 16 h of infection/transfection [26]. Therefore, it may be possible that viruses use different mechanisms to accumulate p53 at the later time point of infection for the induction of cellular stress and apoptosis. Further, we studied the nuclear localization of sp100 and there was no significant change observed in nuclear localization of sp100. The confocal microscopy showed that sp100 protein was still present in the nucleus after NS1 transfection, but there was no co-localization observed with PML nuclear body structure (Fig 3C). Although microscopic data showed the effect of NS1 transfection on the different components of ND10 complex, we performed immunoblot experiment to check the nuclear localization of these proteins at different time points after transfection. With the increase of NS1 expression in the transfected cells, the nuclear localization of Daxx protein decreased gradually (Fig 3D and 3E) while the level of sp100 remained unchanged. The 4 h after transfection, the p53 level was low and the accumulation of p53 initiated after 16 h of transfection that reached its peak value after 48 h of transfection. Yan et al. (2016) showed that the NS1 transfection can increase the p53 level at the later time point (24 h) after transfection [27]. This might be through the inhibition of ubiquitin ligase RNF43 [26]. This alternative mechanism of p53 accumulation could be the one reason of induction of cellular stress and apoptosis. NS1 protein of influenza virus modulates the interaction among the PML, Daxx, sp100 and p53 in such a way that favours the viral replication through p53 inactivation and later it increased p53 accumulation to induce host cell apoptosis in an alternative way. We did not observe any co-localization of p53, sp100 and PML nuclear body in the transfected cells as NS1 induced the degradation of the PML nuclear body (Fig 3D and 3E). Therefore, it can be assumed that NS1 inhibited the localization of p53 within the PML nuclear body at the mid phase -of transfection where p53 can undergo several posttranslational modifications for activation.

### IAV infection induced an oxidative stress and compromised mitochondrial integrity

**Fig 4:**
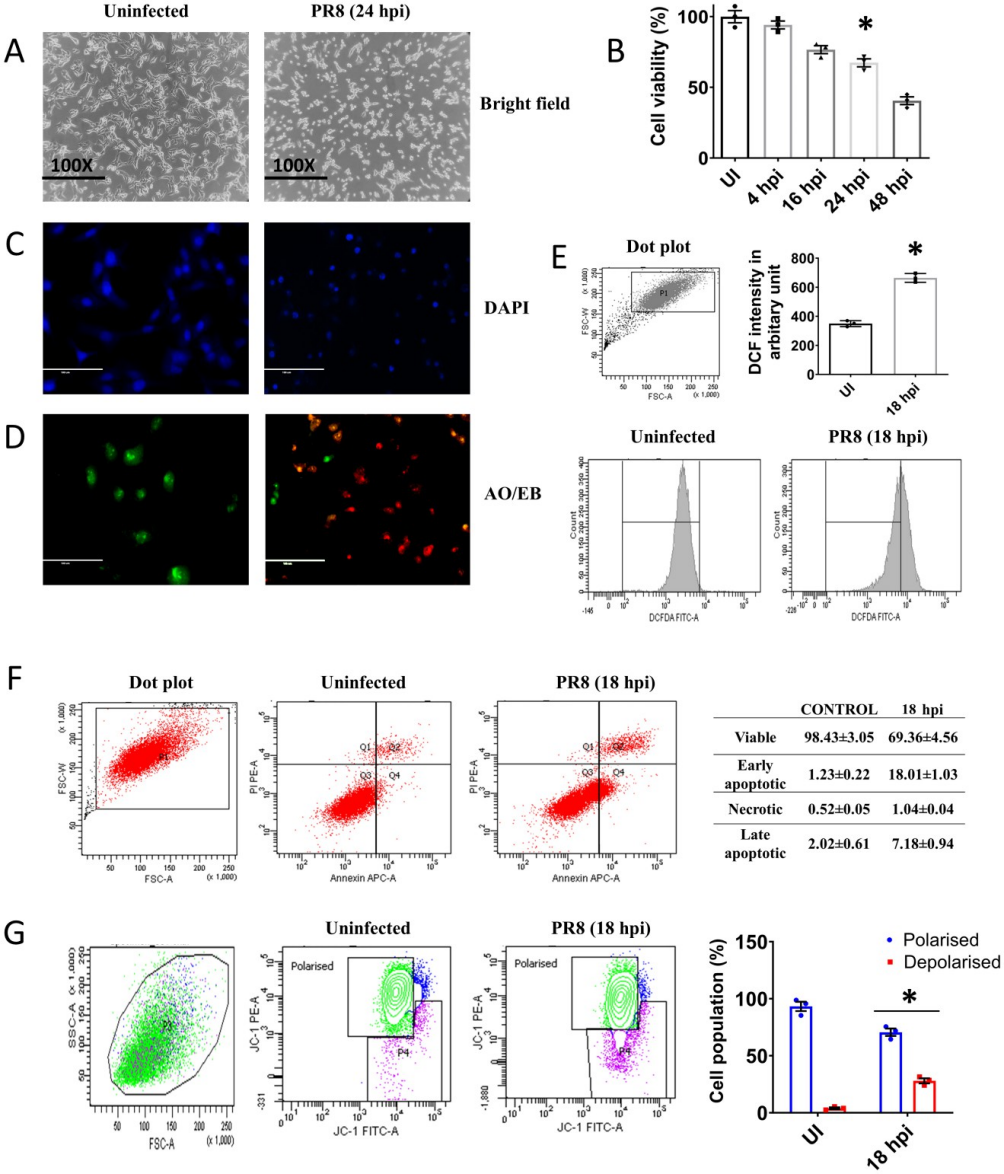
IAV infection induced apoptosis in A549 cells at later phase of infection. **A.** The bright field images show the morphological alteration. **B.** The bar diagram shows the cell viability at different time points after infection. **C.** DAPI staining to observe nuclear damage in infected cells. **D.** AO-EB staining to observe membrane disintegration. **E.** The histogram plot shows the ROS level and bar diagram indicating the DCF fluorescence intensity in different groups. **F.** AnnexinV-APC and PI staining. The Q3 quadrant represents viable cell population, Q4 early apoptotic, Q2 late apoptotic and Q1 necrotic. **G.** JC1 staining to determine MMP. The green population represents the viable cells with no loss of MMP and the purple population stands for the depolarised population with loss of MMP. The values were represented as Mean±SEM (n = 3). p < 0.05 was considered as significant. The significance of differences was calculated between the ‘*’ control vs infection/transfection group.

Degradation of PML nuclear body can lead to the ROS accumulation. Previous literature showed that the degradation of PML nuclear body increased ROS level. Pml^-/-^ cells showed higher ROS accumulation and Pml^-/-^ embryo showed acute glutathione depletion [28]. Therefore, we were intrigued to study the IAV infection induced cellular stress responses when PML was degraded (18 hpi) in the host cells. The reduction in the cell proliferation rate is one of the important parameters for cellular stress response. Thus, the cell viability was measured by MTT assay which showed gradual decrease in cell proliferation with increasing time point after infection. The viability of the control cell was considered as 100%. The cell morphology was also observed under a bright field microscope to observe the morphological alterations after viral infection. The cellular morphology was severely altered in infected cells compared to uninfected one (Fig 4A and 4B).

After IAV infection, A549 cells were stained with Acridine orange–EtBr mixture or DAPI to observe the changes in the cellular morphology at indicated time points. In the infected group, cells appeared rounded with fragmented nuclei as evident from DAPI staining compared to uninfected cells (Fig 4C). The green nuclei represent the viable cells in AO-EtBr staining, yellowish green nuclei stand for early apoptotic population and orange to red nuclei stand for late apoptotic population (Fig 4D). The percentage of late apoptotic and necrotic cell population was significantly (p < 0.05) higher in infected groups compared to control (Fig 4D). As PML degradation can increase the ROS production [28], the ROS generation was measured by flow cytometry using H_2_DCFDA (Fig 4E). The shift of histogram towards the right indicated the higher ROS level in the infected group. The externalization of phosphatidylserine (PS) in the outer leaflet of the bio-membrane and PI incorporation were examined by flow cytometry. The infection group showed higher percentage (p<0.05 was considered as significant) of early apoptotic (18%) and late apoptotic cells (8%) compared to control group (1.23% of early apoptotic cells and 2.02 of late apoptotic cells) (Fig 4F). As excess ROS lead to mitochondrial stress, we measured the mitochondrial membrane potential by JC1. We found that IAV infection significantly (p<0.05) increased the population in Q2 quadrant (22%) (Fig 4G) which was depolarised cell population with loss of membrane potential. The uninfected cells showed a comparatively low percentage (1.67%) of cell population in Q2 quadrant. Therefore, loss of membrane potential after IAV infection was more compared to control.

### The IAV infection induced apoptosis through inhibition of PI3K/AKT and Nrf2 pathways in A549 cells

**Fig 5:**
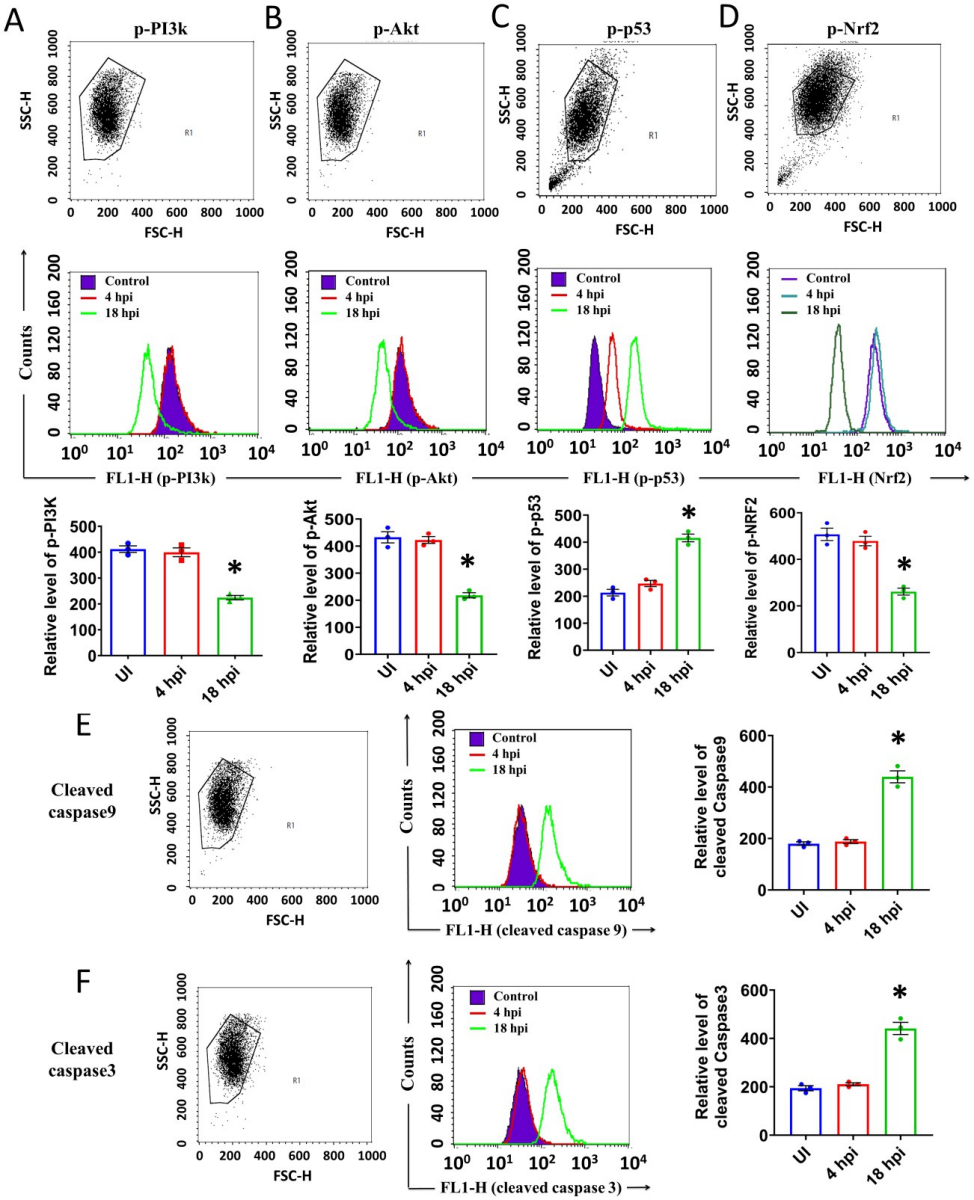
IAV infection induced apoptosis in A549 cells. The expression of **A** p-PI3K, **B** p-Akt, **C** p-53, **D** p-Nrf2, **E** cleaved caspase9, and **F** cleaved caspase3 was measured by flowcytometry in uninfected and IAV infected cells. The counts were plotted along the Y axis and the fluorescence intensity was plotted along the X axis. The bar diagrams represent the intensity of fluorescence in the FL1-H channel. The values were represented as Mean±SEM (n = 3). p < 0.05 was considered as significant. The significance of differences was calculated between the ‘*’ control vs infection/transfection group.

As the IAV infection increased the cellular ROS level in A549 cells through the degradation of PML-NB, we became interested to observe whether this ROS has any impact on the PI3K-Akt signaling pathway and p-Nrf2 level using flowcytometry. After 4 h of infection, there was an increase in phospho-PI3K or phospho-Akt level in A549 cells. But the level was significantly reduced post 18 h of infection (Fig 5A and 5B). We also evaluated the phospho-p53 (phospho Ser15) and phospho-Nrf2 level at the same time points and found an increased level of phospho-p53 (phospho Ser15) with compromised Nrf2 after18h of infection (Fig 5C and 5D). The level of active Caspase-9 and Caspase -3 followed a similar trend. The levels of active Caspase -9 and Caspase- 3 increased in infected cells after 18 h of incubation and it was 2.1 and 2.2 folds higher compared to uninfected cells respectively (Fig 5E and 5F). The results confirmed the cellular apoptosis due to IAV infection at the late phase of infection.

### Influenza virus infection reduced phosphorylated IKKε

**Fig 6:**
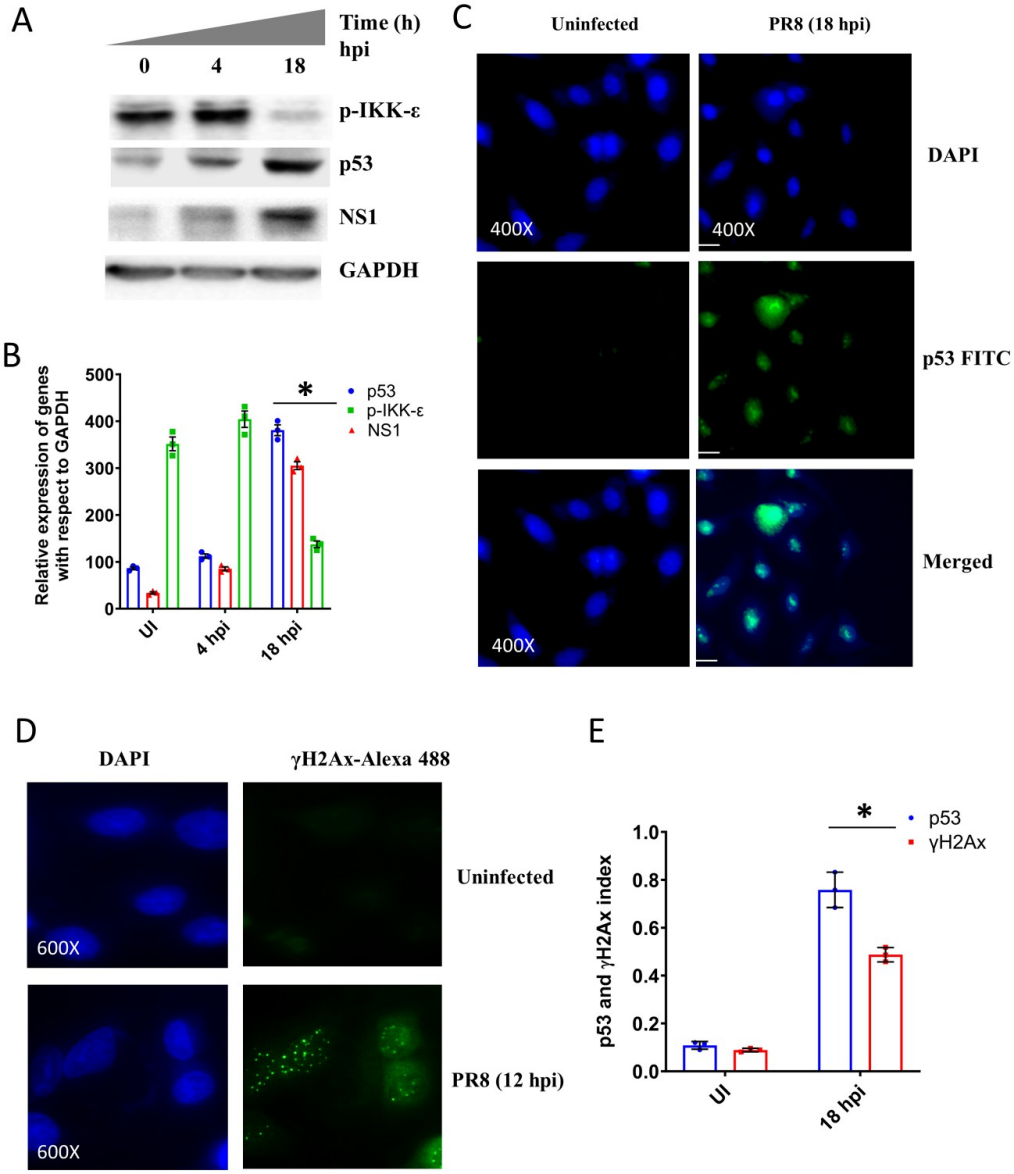
The IAV infection increased γ-H2Ax but prevents IKKε activation. **A.** The immunoblot images show the phosphorylation and expression of different proteins. **B.** The densitometric analysis of immunoblot data. **C.** Immunofluorescence data show nuclear localization of p53 (FITC positive cells) after IAV infection. **D.** Immunofluorescence data show γ-H2Ax focci after 18 hpi. **E.** Quantification of immunofluorescence data of C and D. p < 0.05 was considered as significant. The values were represented as Mean±SEM [(n = 3 for immunoblot) and (n = 3 for microscopic data)]. The significance of differences was calculated between the ‘*’ control vs infection/transfection group.

In this study, we found the IAV infection reduced the expression of the PML nuclear body as seen in immunoblot results while confocal images showed the degradation of the PML nuclear body (Fig 1). We sought to find out the molecular mechanism behind the reduction of the PML level and its degradation after IAV infection. One possible way through which IAV can reduce the PML expression is the blocking of the interferon pathway. It is known that Influenza virus interferes with the host interferon pathway to suppress the inflammatory response for the establishment of viral infection [29]. To check the status of interferon pathway in IAV infected A549 cells, we became intrigued to study the phosphorylation of IRF-3, and STAT1 by immunoblotting at indicated time points (S1 Fig). The activation and phosphorylation of IRF-3, and STAT1 protein increased 4 hpi with IAV. However, a significant decrease in the phosphorylation was observed 18 hpi with IAV (S1 Fig). This indicated that initially host cells tried to increase interferon response to eliminate the virus particle but as virus captured all host machinery and started the propagation, the interferon pathway got inhibited. We also found out another important component of the interferon pathway, IKKε, which has a significant role in PML nuclear body formation. The immunoblot data showed IAV infection reduced the p-IKKε level 18 hpi (Fig 6A and 6B). Previous study showed IKKε in phosphorylated condition entered the nucleus and caused the phosphorylation of PML protein. This phosphorylation helped in PML nuclear body formation and induced PML SUMOylation to recruit sp100, Daxx, p53 in PML nuclear body [30]. Therefore, reduction in phosphorylation of IKKε inhibited its nuclear translocation and inhibited PML nuclear body formation. With the increase of NS1 expression, the interferon pathway got suppressed while the p53 level got increased in infected cells (Fig 6A–6D). It is important to mention here that like the transfection model, IAV infection also showed low level of p53 after 4 h of infection; but this level was increased at the later phase of infection. Shen Y et al. (2009) reported a biphasic pattern of p53 accumulation after IAV infection. They observed the accumulation of p53 after 1–3 h of viral infection, and it was going down in between 4–12 h of post viral infection and again accumulated 14 h of post viral infection [31]. In our study, we did not check the p53 level after 1–3 h of viral infection, but after 4 h we found lesser p53 accumulation and it increased significantly after 18 h of viral infection. Additionally, we found the γ-H2Ax foci formation 18 hpi which indicated the initiation of apoptosis events in infected cells (Fig 6D and 6E).

### NS1 overexpression also reduced the IKKε phosphorylation

**Fig 7:**
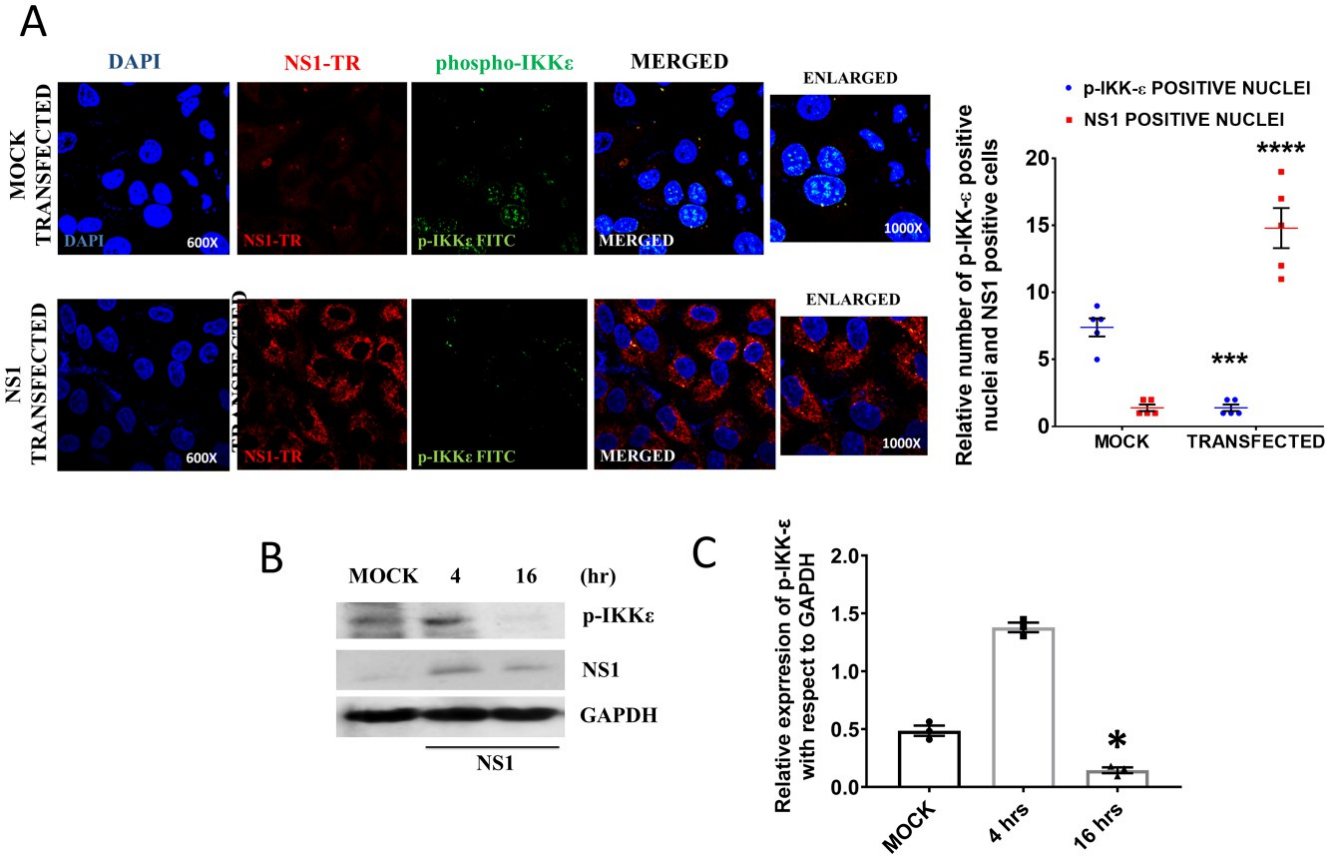
The NS1 overexpression in transfected cells prevents IKKε phosphorylation and nuclear localization. **A.** The confocal images show the phosphorylation level of IKKε. The nuclei were stained by DAPI and p-IKKε was stained by FITC tagged secondary antibody and NS1 was stained by Texas red tagged secondary antibody. The bar diagram represents the number of phospho-IKKε positive nuclei in mock and NS1 transfected cells as observed in 5 independent microscopic data **B.** The immunoblot images showed time dependent levels of p-IKKε and expression of NS1 and transfected cells. **C.** Densitometric analysis of the p-IKKε immunoblot data. p < 0.05 was considered as significant. The values were represented as Mean±SEM [(n = 3 for immunoblot) and (n = 5 for microscopic data)]. The significance of differences was calculated between the ‘*’ control vs infection/transfection group.

As we found a reduced level of p-IKKε when NS1 was expressed in the IAV infection model, we checked the phosphorylation of IKKε in the transfection model. NS1 transfection decreased the phosphorylation of the host IKKε protein. The mock transfected cells showed higher levels of p-IKKε compared to the NS1 transfected cells. The image clearly showed the inhibition of nuclear translocation of IKKε in NS1 transfected cells. The immunoblot data further suggested that initially the NS1 transfection increased the level of p-IKKε. After 16 h of transfection the phosphorylation of IKKε and its nuclear translocation was significantly reduced which corroborated with our infection model (Fig 7A–7C). Similar scenario was observed in the case of the phospho-STAT1 protein. The phosphorylated STAT1 is the active form of STAT1 which acts as a transcription factor for IFN-α/β genes. The mock transfected cells showed higher phospho-STAT1 protein in the nuclei of the cells. However, the level decreased significantly in the NS1 transfection model (S2 Fig) which was further supported by the immunoblot data.

### NS1 overexpression altered the PML SUMOylation pattern

**Fig 8:**
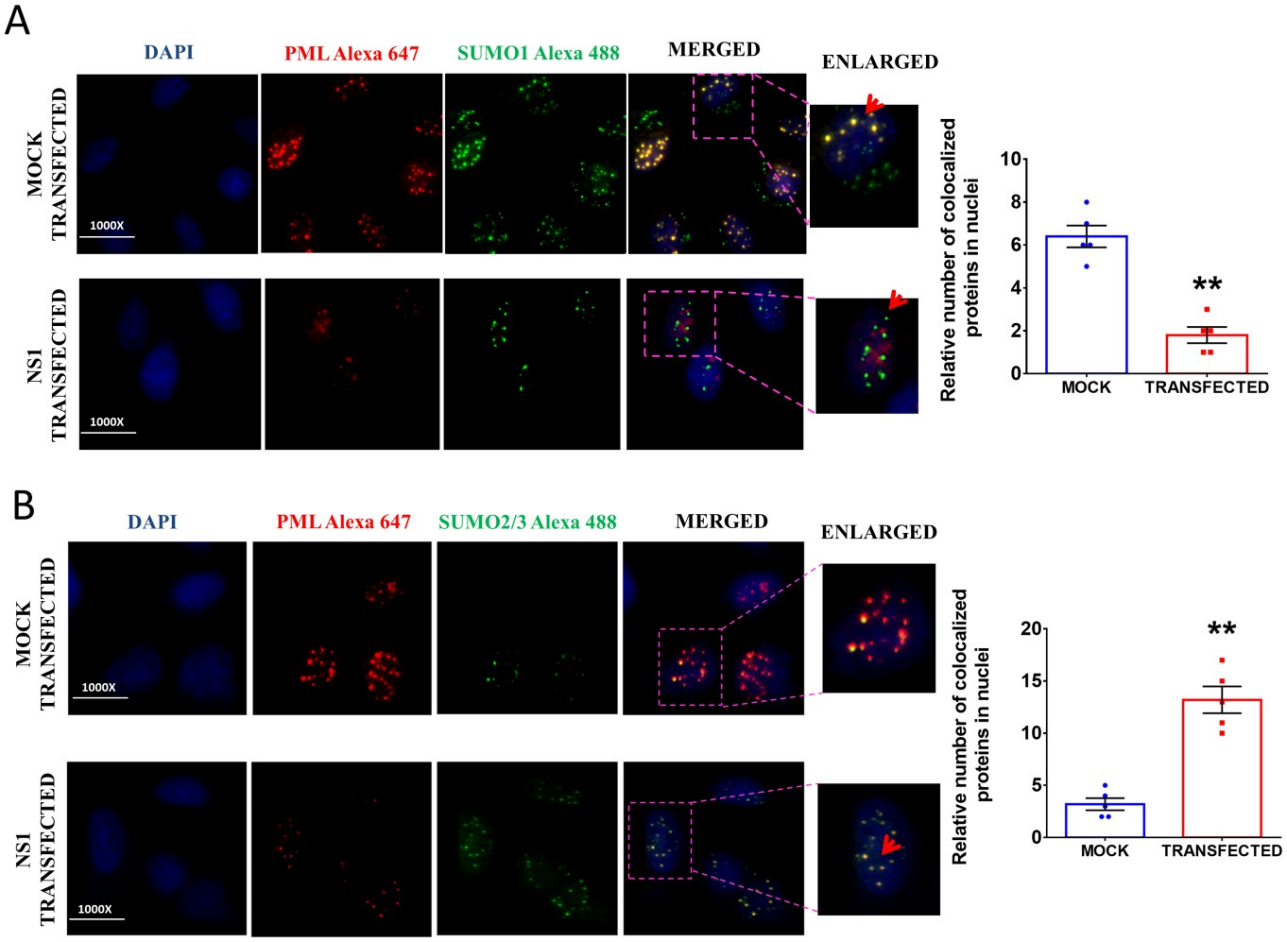
NS1 changes the SUMOylation pattern of PML. The confocal images show the co-localization of **A.** PML with SUMO 1. The bar diagram represents the number of PML and SUMO1 colocalized nuclei in mock and NS1 transfected cells as observed in 5 independent microscopic fields. and **B.** PML with SUMO 2/3 in different groups. The nuclei were stained by DAPI, PML was stained by Alexa 647 and SUMO 1 or SUMO 2/3 was stained by an Alexa 488 tagged secondary antibody. The bar diagram denotes the number of PML and SUMO2/3 colocalized nuclei in mock and NS1 transfected cells as observed in 5 independent microscopic fields. p < 0.05 was considered as significant. The values were represented as Mean±SEM (n = 5).

Since p-IKKε has a role in the SUMOylation of the PML nuclear body, we became interested in checking SUMOylation pattern of PML to understand the reason of destabilization of host ND10 complex which comprises of PML, sp100, Daxx and p53. After NS1 transfection, we assessed the SUMOylation pattern of PML. The present experimental data showed NS1 transfection decreased the SUMO 1 conjugation to the PML nuclear body while most of the PML nuclear body remained conjugated with SUMO 1 in the mock transfected cells (Fig 8A). NS1 induced the SUMO 2/3 conjugation to PML nuclear body (Fig 8B) which led to the degradation of PML nuclear body and thereby impeded the formation of host ND10 antiviral complex.

## Discussion

In this study, we highlight the role of NS1 of influenza A virus in escaping the host antiviral response via disruption of ND10 nuclear body structure which is alternatively known as PML nuclear bodies (PML-NB). We tried to identify the molecular mechanism by which NS1 degraded PML-NB followed by the formation of ND10 complex. The PML nuclear body is associated with diverse functions, and it is heterogeneous in terms of its structure and function [32, 33]. Though PML nuclear bodies are targeted by viral proteins NS1 during Influenza A virus infection, it does not localize to PML nuclear bodies [18]. Moreover, the mechanism of how NS1 inhibits the PML-NB formation remains unidentified. Earlier reports have shown that NS1 protein interferes with the host interferon pathway to establish the viral infection [34].

**Fig 9:**
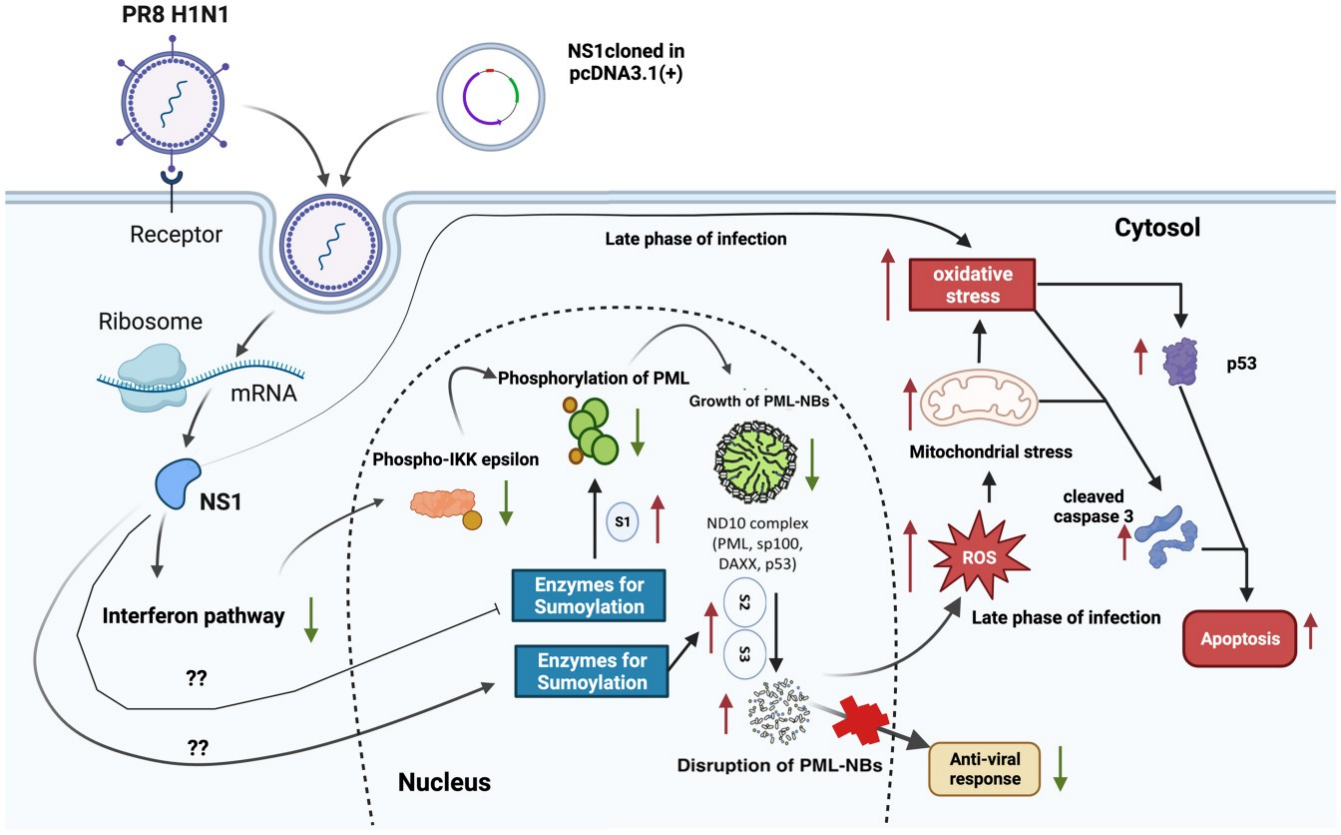
The schematic representation of overall outcome of the study. IAV infection/NS1 transfection alters the interferon pathway and IKKε phosphorylation which inhibits PML-NB formation. NS1 also changes the SUMOylation pattern of PML-NB which leads to its degradation. PML-NB degradation increases the oxidative stress through elevated level of ROS at the late phase of infection. On the top of that IAV infection and NS1 transfection both generate cellular stress at late time points and induce oxidative stress. ROS alters the mitochondrial function and activates several stress signalling pathways which at the end activate p53 and caspase 3 to induce apoptosis.

Literature survey revealed that the phosphorylation of IKKε and its nuclear localization is a prerequisite for the formation and retention of PML-NB [30]. Interestingly, there was a decrease in the phosphorylation of IKKε (18 hpi) observed when infected cells showed strong expression of NS1. Moreover, this phosphorylation and nuclear localization of IKKε was also inhibited in NS1 transfected A549 cells. Therefore, our data revealed a critical role of NS1 in preventing the PML-NB formation via inhibition of IKKε activation. The host ND10 complex is composed of various cellular proteins like PML, Daxx, sp100, p53 etc. All these proteins associate in PML-NB to form functional ND10 complex. As the synthesis of PML protein was greatly hampered by viral NS1 protein, it eventually inhibited the whole ND10 complex formation. There was no co-localization of sp100 within the PML-NB observed in NS1 transfected cells. In addition to that, Daxx was also present in the cytosol which otherwise localized in the nucleus of the mock transfected cells. All these findings clearly described disintegration of ND10 antiviral complex. In both the IAV infection and NS1 transfection model, an increase in nuclear localization of p53 was observed at the late phase (after 18 h in infection and 16 h in transfection model) but not in early phase (4 h). Previous report reveals the activation of p53 occurs in a biphasic way after IAV infection. The level of p53 increases after 1 h of viral infection followed by reduction after 4 h which again increases after 18 h [31]. This result corroborated with our findings. A possible explanation is initially when virus infects one cell, it breaks the host DNA which causes the p53 accumulation. To finish the viral replication, virus temporarily inhibits the p53 accumulation and activation. After finishing the replication, it allows the generation of cellular stress, ROS production, p53 accumulation, and all other apoptotic events to induce host cell death. Both the IAV infection and NS1 transfection induced the degradation of PML-NB as a result of which p53 could not co-localize with PML-NB. This inhibits post-translational modification and activation of p53 at the mid phase of infection which may help virus to finish its replication process keeping host cell alive. At the late phase of infection, viruses use different mechanisms to increase p53 level and induce cellular stress [26]. Additionally, PML degradation boosted the ROS level and generated oxidative stress. All these factors together led the mitochondrial dysfunction and induced the apoptosis of the host cell to release mature virus particles (Fig 9).

To find out the mechanism of PML degradation, we explored the detailed structure of PML-NB. Literatures survey reveals that the PML contain numerous protein‐modifying enzymes for post-translational protein modifications [35] and they are considered as cellular hotspots of SUMOylation (posttranslational conjugation with the small ubiquitin‐like modifier (SUMO)) [36, 37]. Influenza viruses were reported to interact extensively with the host SUMOylation system. They cause a global change of cellular SUMOylation pattern, even though overall protein synthesis is downregulated [38]. Therefore, in the notion of these evidence, we evaluated the SUMOylation pattern of PML-NB in mock and NS1 transfected cells. In the mock transfected cells, most of the PML remained SUMO-1 conjugated while NS1 transfection changed it to SUMO-2/3. It was already established that PML is critical for ND10 formation and recruits the PML-interacting proteins when modified by SUMO-1 [39] while the SUMO-2/3 conjugation of PML initiates the RNF4 dependent disruption of PML-NB [40, 41]. Thus, the SUMO-2/3 conjugation of PML-NB in NS1 transfected condition disrupted the ND10 formation. Our results help to understand the crucial role of multi-functional protein of Influenza A virus NS1 in inhibiting Interferon pathway involving PML and its associated proteins in PML-NBs. The importance of p-IKKε in PML-NBs formation and changes in the SUMOylation pattern was studied. Thus, the present study highlighted a host-virus interaction among NS1-PML-SUMO for the disruption of PML-NBs formation (Fig 9) in order to inhibit the antiviral strategy of the hosts.

In conclusion, we established a prominent role of NS1, a highly conserved and multifunctional protein of IAV, in establishing viral infection through disruption of ND10 complex formation. It strengthens the idea for discovery of anti influenza targets for drug development. Therefore, NS1 can be a potential drug target for eliminating seasonal spread of influenza virus infection though, it necessitates further detailed investigation.

## Supporting information

S1 Fig Prevention of interferon pathway activation in A549 cells infected with IAV: A. The immunoblot images showed time-dependent levels of p-IRF3 and p-STAT1 in IAV infected cells. B. The densitometric analysis of immunoblot data.
(PDF)

S2 Fig Prevention of interferon pathway activation in A549 cells transfected with NS1: A. The confocal images show the phosphorylation level of STAT1. The nuclei were stained by DAPI and p-STAT1 was stained by FITC tagged secondary antibody. NS1 was stained by texas red tagged secondary antibody. B. The immunoblot images showed time-dependent levels of p-STAT1in NS1 transfected cells. C. The densitometric analysis of immunoblot data.
(PDF)

S1 Table Individual data points for all biological replicates. (PDF)
