# Supplementary material for: Role of Influenza A virus protein NS1 in regulating host nuclear body ND10 complex formation and its involvement in establishment of viral pathogenesis
Source: PLoS One. 2024 Jan 2;19(1):e0295522. doi: 10.1371/journal.pone.0295522 (PMC10760828; doi:10.1371/journal.pone.0295522)
Supplement: S1 Fig — Prevention of interferon pathway activation in A549 cells infected with IAV: A. The immunoblot images showed time-dependent levels of p-IRF3 and p-STAT1 in IAV infected cells. B. The densitometric analysis of immunoblot data. (PDF) [file pone.0295522.s001.pdf]

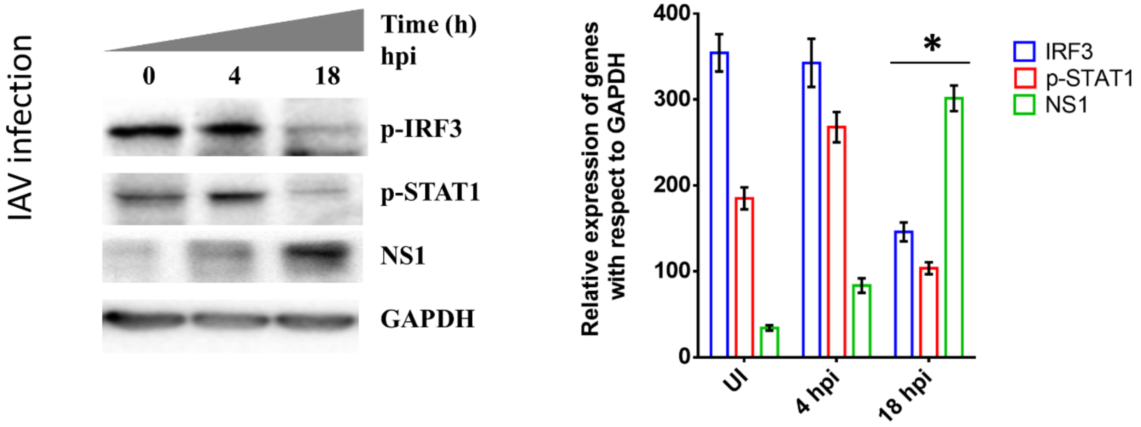

S1 fig: Prevention of interferon pathway activation in A549 cells infected with IAV: A. The immunoblot images showed time-dependent levels of p-IRF3 and p-STAT1 in IAV infected cells. **B.** The densitometric analysis of immunoblot data.
