# Supplementary material for: Role of Influenza A virus protein NS1 in regulating host nuclear body ND10 complex formation and its involvement in establishment of viral pathogenesis
Source: PLoS One. 2024 Jan 2;19(1):e0295522. doi: 10.1371/journal.pone.0295522 (PMC10760828; doi:10.1371/journal.pone.0295522)
Supplement: S2 Fig — Prevention of interferon pathway activation in A549 cells transfected with NS1: A. The confocal images show the phosphorylation level of STAT1. The nuclei were stained by DAPI and p-STAT1 was stained by FITC tagged secondary antibody. NS1 was stained by texas red tagged secondary antibody. B. The immunoblot images showed time-dependent levels of p-STAT1in NS1 transfected cells. C. The densitometric analysis of immunoblot data. (PDF) [file pone.0295522.s002.pdf]

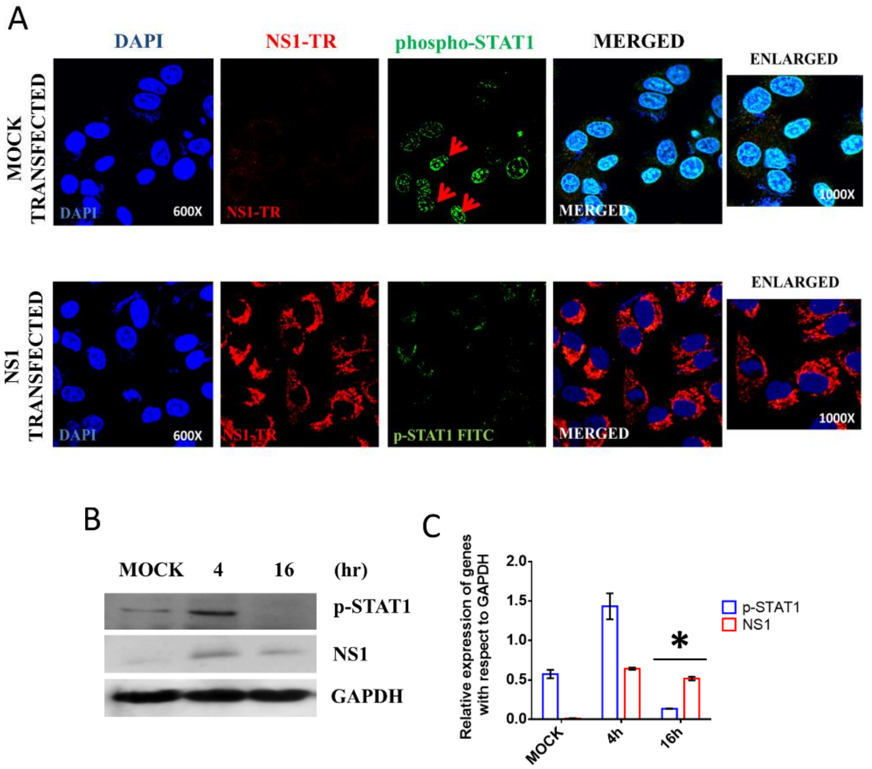

S2 fig: Prevention of interferon pathway activation in A549 cells transfected with NS1: A. The confocal images show the phosphorylation level of STAT1. The nuclei were stained by DAPI and p-STAT1 was stained by FITC tagged secondary antibody. NS1 was stained by texas red tagged secondary antibody. **B.** The immunoblot images showed time-dependent levels of p-STAT1in NS1 transfected cells. **C.** The densitometric analysis of immunoblot data.
