# Supplementary material for: Role of Influenza A virus protein NS1 in regulating host nuclear body ND10 complex formation and its involvement in establishment of viral pathogenesis
Source: PLoS One. 2024 Jan 2;19(1):e0295522. doi: 10.1371/journal.pone.0295522 (PMC10760828; doi:10.1371/journal.pone.0295522)
Supplement: S1 Table — (PDF) [file pone.0295522.s003.pdf]

S1 Table: Individual data points for all biological replicates

	IF=Immunofluorescence											
	WB=Western blot											
1	PML, NS1 (Infection) WB		PML	PML	PML	NS1	NS1	NS1				
		UI	340.87	367.595	318.478	34.057	25.585	42.847				
		4 hpi	360.223	380.663	334.223	136.527	154.484	167.847				
		18 hpi	82.383	71.868	74.988	301.489	335.747	272.473				
2	PML NB/cell (Infection) IF	Uninfected	PR8 (18 hpi)									
		9	3									
		14	5									
		17	4									
		8	2									
		10	6									
3	NS1 positive cell (Transfection) IF	MOCK	TRANSFECTED									
		2	15									
		3	18									
		1	12									
		1	9									
		2	13									
4	NS1(transfection)WB	MOCK	4 hrs	16 hrs	48 hrs							
		21.08	152.81	355.237	367.32							
		23.745	173.479	380.658	396.786							
		18.447	132.757	330.465	337.53							

5	PML (Transfection) WB		PML III	PML III	PML III	PML I/II/IV	PML I/II/IV	PML I/II/IV	PML+SUMO	PML+SUMO	PML+SUMO	
		MOCK	385.346	393.675	369.555	233.633	246.866	225.669	547.272	556.474	534.554	
		4 hrs	321.635	332.756	314.559	173.536	182.217	164.977	468.626	476.378	452.836	
		16 hrs	112.473	118.373	106.336	35.843	38.484	30.373	215.222	222.363	207.338	
		48 hrs	91.07	86.373	96.228	32.462	27.773	35.447	250.692	253.448	244.364	
6	PML NB/cell and NS1 positive cells (Transfection) IF		PML POSITIVE NUCLEI					NS1 POSITIVE CELLS				
		MOCK	6	11	9	8	13	0	1	1	2	2
		TRANSFECTED	2	4	1	3	3	12	14	9	15	13
			NS1 POSITIVE CELLS					DAXX POSITIVE NUCLEI				
7	Daxx and NS1 (Transfection) IF	MOCK	1	2	1	2	1	7	8	9	10	12
		TRANSFECTED	15	17	14	12	9	1	3	2	4	2
8	p53 and PML/cell (Transfection) IF		PML POSITIVE NUCLEI					p53 POSITIVE NUCLEI				
		MOCK	8	7	8	6	10	1	0	1	0	1
		TRANSFECTED	2	1	4	3	2	4	5	6	4	5
9	sp100 and PML/cell (Transfection) IF	MOCK	TRANSFECTED									
		12	1									
		8	2									
		15	2									
		10	3									

		7	1								
10	sp100, Daxx, p53 (Transfection) WB		sp100	sp100	sp100	DAXX	DAXX	DAXX	p53	p53	p53	
		MOCK	340.746	356.879	326.997	638.73	662.764	616.554	55.24	62.511	49.833	
		4 hrs	389.57	403.734	377.966	521.24	542.488	503.623	48.66	44.786	51.63	
		16 hrs	310.938	332.668	291.441	301.378	319.487	286.227	189.33	204.256	174.661	
		48 hrs	307.11	324.755	292.114	120.839	109.35	133.768	412.32	388.22	436.687	
11	Cell viability (Infection) MTT	UI	4 hpi	16 hpi	24 hpi	48 hpi						
		100	94.373	80.372	68.373	35.538						
		107.273	98.739	71.122	61.937	41.037						
		92.372	89.113	78.339	71.554	44.937						
12	ROS (Infection) Flowcytometry	UI	18 hpi									
		350.749	635.584									
		328.584	695.794									
		369.547	660.473									
			Polarised	Polarised	Polarised	Depolarised	Depolarised	Depolarised				
12	MMP (Infection) Flowcytometry	UI	85.373	98.746	95.663	5.374	2.484	3.958				
		18 hpi	64.484	71.883	75.473	31.474	28.499	24.074				
13	p-PI3K (Infection) Flowcytometry	UI	4 hpi	18 hpi								
		411.727	402.372	224.271								
		389.773	368.477	209.483								

		433.927	427.378	238.746								
14	p-Akt (Infection) Flowcytometry	UI	4 hpi	18 hpi								
		433.557	417.935	212.574								
		467.877	402.977	204.757								
		395.866	446.457	236.588								
15	p-p53 (Infection) Flowcytometry	UI	4 hpi	18 hpi								
		216.48	254.292	416.489								
		190.373	261.836	390.383								
		232.473	224.383	439.626								
16	p-Nrf2 (Infection) Flowcytometry	UI	4 hpi	18 hpi								
		502.843	470.11	267.493								
		555.383	517.337	232.879								
		463.478	448.4747	283.675								
17	cleaved Caspase9 (Infection) Flowcytometry	UI	4 hpi	18 hpi								
		189.304	200.384	435.843								
		166.675	188.875	481.373								
		183.756	174.654	401.474								

18	cleaved Caspase3 (Infection) Flowcytometry	UI	4 hpi	18 hpi								
		197.483	210.77	445.239								
		174.437	199.373	482.474								
		210.373	220.373	395.883								
19	p53,p-IKK-ε, NS1 (Infection) WB		p53	p53	p53	p-IKK-ε	p-IKK-ε	p-IKK-ε	NS1	NS1	NS1	
		UI	80.867	90.878	86.677	350.87	377.595	326.478	34.057	29.585	37.847	
		4 hpi	120.755	104.868	110.785	410.223	430.663	371.223	83.527	78.484	92.847	
		18 hpi	400.633	380.757	360.688	149.383	124.868	137.988	301.489	320.747	292.473	
20	p53, γH2Ax (Infection) IF		p53	p53	p53	γH2Ax	γH2Ax	γH2Ax				
		UI	0.1203	0.1153	0.0903	0.089	0.096	0.081				
		18 hpi	0.758	0.832	0.6845	0.486	0.518	0.458				
21	p-IKK-ε (Transfection) IF	Ikk-ε POSITIVE NUCLEI					NS1 POSITIVE NUCLEI					
		8	7	9	8	5	1	1	2	1	2	
		1	2	2	1	1	15	17	19	12	11	
22	p-IKK-ε (Transfection) WB	MOCK	4 hrs	16 hrs								
		0.4824	1.384	0.15743								
		0.5641	1.448	0.17973								
		0.41121	1.305	0.09573								

23	PML-SUMO1/cell (Transfection) IF	MOCK	TRANSFECTED									
		8	1									
		6	2									
		7	2									
		6	3									
		5	1									
24	PML-SUMO2-3/cell (Transfection) IF	MOCK	TRANSFECTED									
		2	10									
		3	15									
		4	11									
		2	13									
		5	17									
